# P38α-MAPK Signaling Inhibition Attenuates Soleus Atrophy during Early Stages of Muscle Unloading

**DOI:** 10.3390/ijms21082756

**Published:** 2020-04-15

**Authors:** Svetlana P. Belova, Ekaterina P. Mochalova, Tatiana Y. Kostrominova, Boris S. Shenkman, Tatiana L. Nemirovskaya

**Affiliations:** 1 Institute of Biomedical Problems, RAS, Moscow 123007, Russia; swetbell@mail.ru (S.P.B.); mochalova_ekaterina@lenta.ru (E.P.M.); bshenkman@mail.ru (B.S.S.); 2 Department of Anatomy, Cell Biology and Physiology, Indiana University School of Medicine-Northwest, Gary, IN 46408, USA; tkostrom@iun.edu

**Keywords:** muscle unloading, MuRF1, MAFbx, p38α-MAPK, calpain-1, ubiquitin

## Abstract

To test the hypothesis that p38α-MAPK plays a critical role in the regulation of E3 ligase expression and skeletal muscle atrophy during unloading, we used VX-745, a selective p38α inhibitor. Three groups of rats were used: non-treated control (C), 3 days of unloading/hindlimb suspension (HS), and 3 days HS with VX-745 inhibitor (HSVX; 10 mg/kg/day). Total weight of soleus muscle in HS group was reduced compared to C (72.3 ± 2.5 vs 83.0 ± 3 mg, respectively), whereas muscle weight in the HSVX group was maintained (84.2 ± 5 mg). The expression of muscle RING-finger protein-1 (MuRF1) mRNA was significantly increased in the HS group (165%), but not in the HSVX group (127%), when compared with the C group. The expression of muscle-specific E3 ubiquitin ligases muscle atrophy F-box (MAFbx) mRNA was increased in both HS and HSVX groups (294% and 271%, respectively) when compared with C group. The expression of ubiquitin mRNA was significantly higher in the HS (423%) than in the C and HSVX (200%) groups. VX-745 treatment blocked unloading-induced upregulation of calpain-1 mRNA expression (HS: 120%; HSVX: 107%). These results indicate that p38α-MAPK signaling regulates MuRF1 but not MAFbx E3 ligase expression and inhibits skeletal muscle atrophy during early stages of unloading.

## 1. Introduction

Skeletal muscle has a large amount of plasticity, responding to the decrease in activity caused by a variety of conditions (immobilization, microgravity, prolonged bed rest, denervation, etc.) by atrophy, declined muscle fiber diameter, loss of protein content, decreased specific force, and increased fatigability [1,2,3]. Skeletal muscle atrophy is caused by accelerated protein degradation and decreased protein synthesis [4,5]. In order to prevent muscle atrophy, it is critically important to understand the molecular mechanisms regulating the initiation of muscle atrophy. The expression of muscle-specific E3 ubiquitin ligases muscle atrophy F-box (MAFbx) and muscle RING-finger protein-1 (MuRF1) is regulated via activity of the ubiquitin–proteasome pathway. The majority of previous studies focused on the role of Forkhead box protein O (FoxO) phosphorylation/de-phosphorylation by Akt in the regulation of E3 ubiquitin ligase expression [6]. Nevertheless, our previous studies showed that during unloading FoxO3 and NF-κB pathways do not inevitably control the upregulation of MuRF1 [7,8,9]. In some instances, different regulatory mechanisms can play the most critical role in the regulation of MuRF1 expression.

It was previously reported that denervation-induced decrease in skeletal muscle mass is regulated via p38α MAP kinase signaling [10]. However, it is not well defined as to whether this signaling pathway also participates in the unloading-induced upregulation of E3 ubiquitin ligases and in the onset of skeletal muscle atrophy. There are three major MAP kinase signaling pathways regulated via phosphorylation: extracellular signal-related kinase (ERK), c-Jun NH2-terminal kinase (JNK), and p38 MAP kinase. p38 MAP kinase plays a major role in the regulation of transcription and in cell motility [11,12]. Only a few studies have looked at the role of p38 MAP kinase during unloading. It was reported that unloading-increased activation of p38 MAP kinase correlated with skeletal muscle atrophy [13,14]. Activation of MAP kinase regulates proliferation and differentiation via phosphorylation of cytoplasmic and nuclear proteins, as well as modulation of gene expression [14]. Dupont and colleagues suggested that increased phosphorylation of p38 MAP kinase after 14 days of unloading can be involved in the regulation of myosin heavy chain expression and unloading-induced changes in myosin composition [14]. It was previously reported that unloading-induced activation of p38 MAP kinase participates in the initiation of protein degradation [15]. Inhibition of p38 MAP kinase activity in cultured muscle cells using SB203580, an inhibitor of p38 MAP kinase, or curcumin decreased activity of ubiquitin. Therefore, p38 MAP kinase may be involved in the regulation of the ubiquitin–proteasome pathway.

Increased expression of E3 ubiquitin ligases during unloading can be detected at the first day, and it peaks by the third day [16]. However, previous studies investigated later time points in p38 MAP kinase activation during unloading. The only exception is the study by Kawamoto and colleagues that showed increased p38 MAP kinase phosphorylation at 6 hours after unloading of rat soleus muscle [17]. Expression of E3 ubiquitin ligases was not tested in this study.

In the current study, we examined whether activation of p38α MAP kinase at the early stages of unloading regulated the expression of muscle-specific E3 ubiquitin ligases MAFbx and MuRF1. We used VX-745, a specific inhibitor of p38α MAP kinase, and tested its effect on the regulation of the ubiquitin–proteasome pathway and on skeletal muscle atrophy during unloading. We hypothesized that blocking of p38α MAP kinase activation will prevent the upregulation of E3 ubiquitin ligases and muscle atrophy during early stages of unloading.

## 2. Results

### 2.1. The Effect of VX-745 Treatment on Muscle Atrophy and Unloading-Induced Protein Degradation Pathways in Rat Soleus Muscle

**Figure 1:**
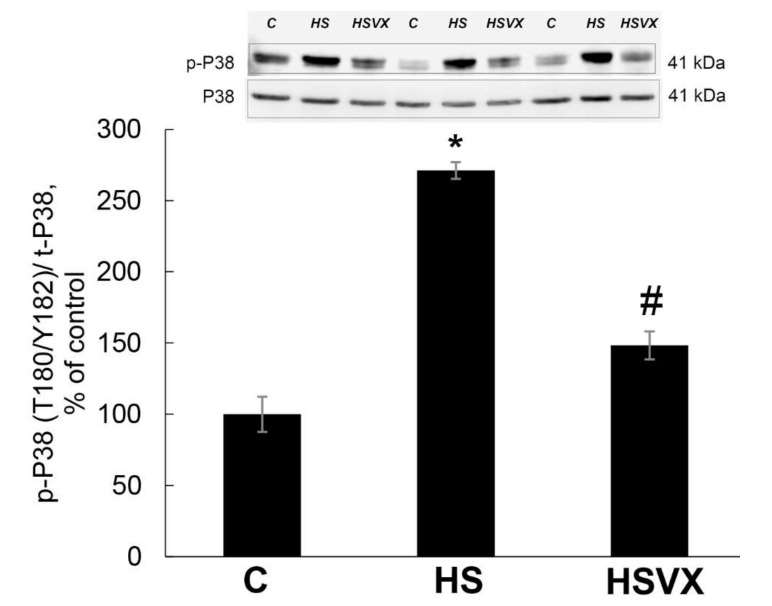
Evaluation of phospho-p38 content in soleus muscles of non-treated control (C), 3 days of unloading/hindlimb suspension (HS), and 3 days HS with VX-745 inhibitor (HSVX) rats by Western blotting. Values are normalized to the levels of total protein and total-p38 expression in each sample. *n* = 8. * indicates a significant difference from the control, *p* < 0.05; # indicates a significant difference from the HS, *p* < 0.05.

To evaluate the effectiveness of VX-745 inhibitor in our experiments, we first determined the level of p38 phosphorylation in soleus muscles of non-treated control (C), 3 days of unloading/hindlimb suspension (HS), and 3 days HS with VX-745 inhibitor (HSVX) rats (Figure 1). Unloading significantly increased the content of phospho-p38 (Figure 1). This increase was blocked by VX-745 treatment (Figure 1). These results confirm the specificity and effectiveness of VX-745 inhibitor in our experimental conditions.

**Figure 2:**
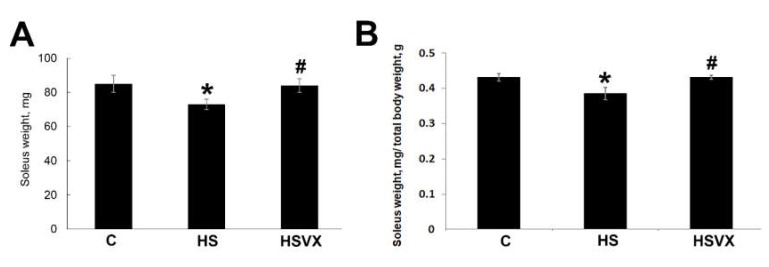
Absolute (**A**) and normalized (**B**) weight of soleus muscles of C, HS, and HSVX rats. *n* = 8. * indicates a significant difference from the control, *p* < 0.05, # indicates a significant difference from the HS, *p* < 0.05.

Three days of unloading had no statistically significant effect on the total body weight of the experimental rats. The average total body weights were 200 ± 13.5, 186 ± 9.97, and 195 ± 12.9 g for C, HS, and HSVX rats, respectively. Treatment with VX-745 prevented unloading-induced soleus muscle atrophy (Figure 2). VX-745 treatment blocked decrease of both absolute soleus muscle mass (Figure 2A) as well as soleus muscle mass normalized to the total body weight (Figure 2B).

**Figure 3:**
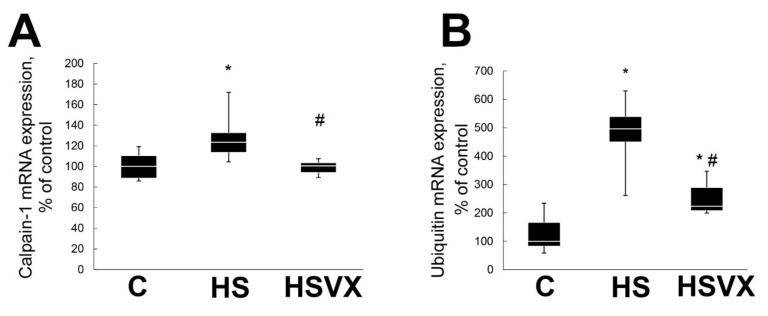
Evaluation of mRNA expression of calpain-1 (**A**) and ubiquitin (**B**) in soleus muscles of C, HS, and HSVX rats. Values are normalized to the level of GAPDH mRNA expression in each sample. *n* = 8. * indicates a significant difference from the control, *p* < 0.05; # indicates a significant difference from the HS, *p* < 0.05.

It is known that, during unloading, calpain-1 and ubiquitin are involved in skeletal muscle protein degradation [9]. As expected, unloading significantly increased calpain-1 mRNA expression, whereas VX-745 treatment blocked this increase (Figure 3A). Similarly, VX-745 treatment diminished unloading-induced upregulation of the ubiquitin mRNA expression (Figure 3B).

**Figure 4:**
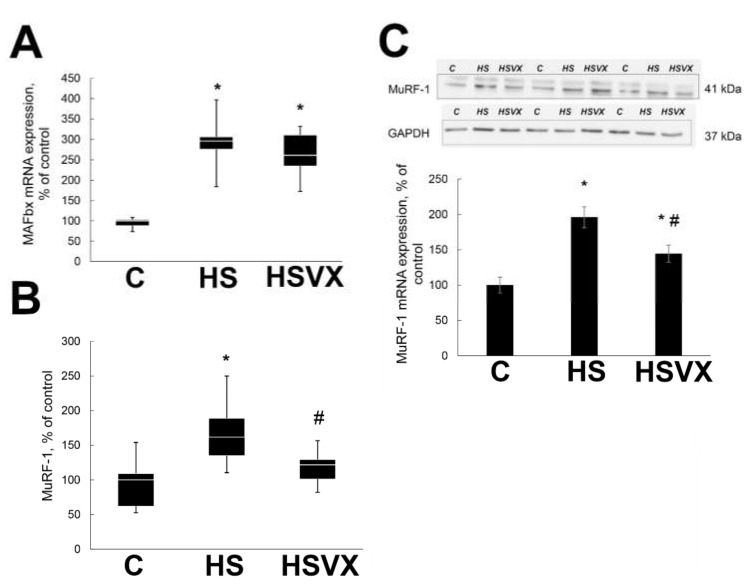
Expression of muscle-specific E3 ubiquitin ligases muscle atrophy F-box (MAFbx) mRNA (**A**) and muscle RING-finger protein-1 (MuRF1) mRNA (**B**) and protein (**C**) in soleus muscles of C, HS, and HSVX rats. (**A**,**B**) Values are normalized to the level of GAPDH mRNA expression in each sample. (**C**) Values are normalized to the levels of total protein and GAPDH protein content in each sample. *n* = 8. * indicates a significant difference from the control, *p* < 0.05; # indicates a significant difference from the HS, *p* < 0.05.

VX-745 treatment differently affected the expression of two skeletal muscle-specific E3 ligases MuRF1 and MAFbx. VX-745 treatment had no effect on the unloading-induced increase in mRNA expression of MAFbx (Figure 4A). At the same time, it significantly diminished the unloading-induced increase in mRNA (Figure 4B) and protein (Figure 4C) expression of MuRF1.

**Figure 5:**
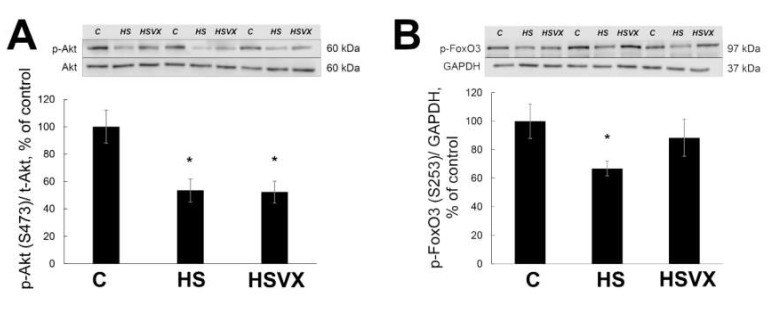
Evaluation of phospho-Akt (**A**) and phospho-FoxO3 (**B**) content in soleus muscles of C, HS, and HSVX rats by Western blotting. Values are normalized to the levels of total protein and total Akt or GAPDH content in each sample. *n* = 8. * indicates a significant difference from the control, *p* < 0.05; # indicates a significant difference from the HS, *p* < 0.05.

**Figure 6:**
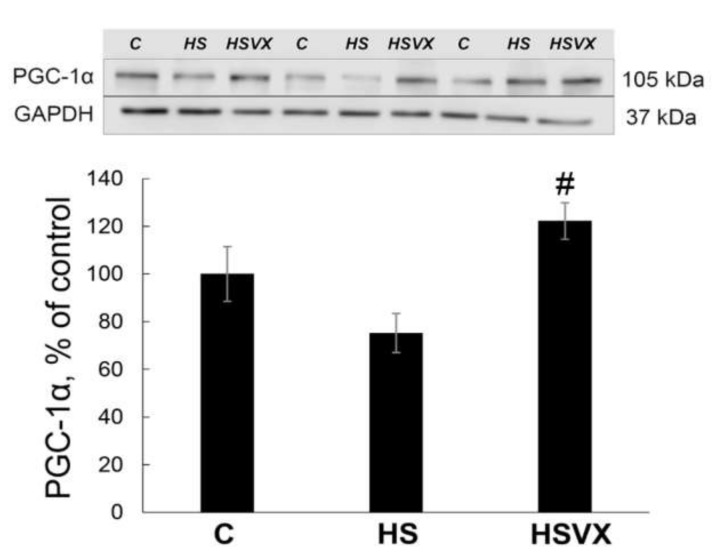
Evaluation of PGC-1α protein expression in soleus muscles of C, HS, and HSVX rats by Western blotting. Values are normalized to the level of GAPDH content in each sample. *n* = 8. # indicates a significant difference from the HS, *p* < 0.05.

It was previously reported that upon muscle unloading, changes in Akt1, FoxO3 [9], and PGC-1α [18] signaling promote protein degradation via induced expression of the muscle-specific ubiquitin ligases MuRF1 and MAFbx. VX-745 treatment had no effect on the unloading-induced decrease in phospho-Akt content (Figure 5A). At the same time, VX-745 treatment blocked unloading-induced decrease in phospho-FoxO3 content (Figure 5B). VX-745 prevented unloading-induced decrease in PGC-1α, and even showed a trend towards PGC-1α content increase when compared with the control muscle (Figure 6).

**Figure 7:**
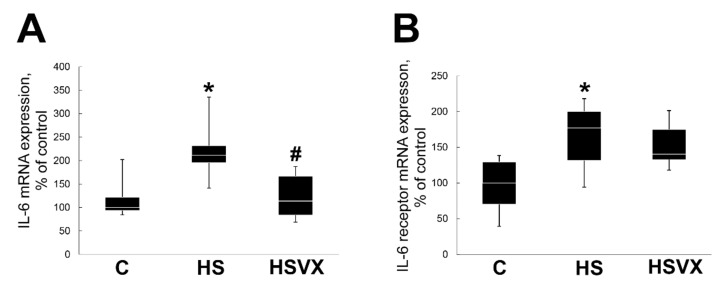
Evaluation of mRNA expression of IL-6 (**A**) and IL-6 receptor (**B**) in soleus muscles of C, HS, and HSVX rats. Values are normalized to the level of GAPDH mRNA in each sample. *n* = 8. * indicates a significant difference from the control, *p* < 0.05; # indicates a significant difference from the HS, *p* < 0.05.

PGC-1α expression is regulated by IL-6 signaling [19]. To test whether increased expression of PGC-1α in the HSVX group correlates with increased IL-6 expression in muscle, we evaluated mRNA expression of IL-6 and IL-6 receptor. In the HS group, expression of both IL-6 and IL-6 receptor were significantly increased (Figure 7). VX-745 treatment blocked unloading-induced IL-6 upregulation, but had no effect on the increased expression of IL-6 receptor (Figure 7).

### 2.2. The Effect of VX-745 Treatment on Protein Synthesis Pathways in Rat Soleus Muscle

**Figure 8:**
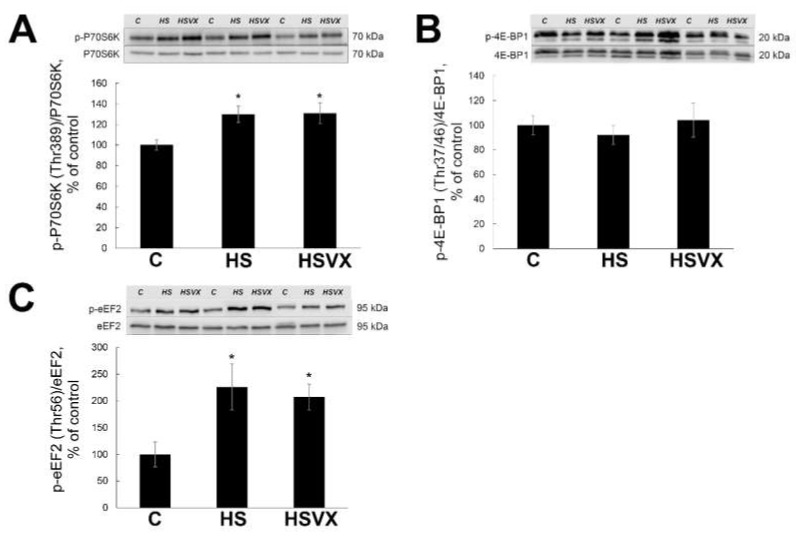
Evaluation of phospho-p70S6K (**A**), phospho-4EBP1 (**B**), and phospho-eEF2 (**C**) content in soleus muscles of C, HS, and HSVX rats by Western blotting. Values are normalized to the levels of total protein and total p70S6K, 4E-BP1, and eEF2 content in each sample. *n* = 8. * indicates a significant difference from the control, *p* < 0.05.

Unloading-induced muscle atrophy is regulated not only via accelerated protein degradation but also by decreased protein synthesis. Therefore, we evaluated the effect of VX-745 treatment on the protein synthesis during unloading. Levels of phospho-p70S6K were significantly increased in both HS and HSVX groups when compared with the control (Figure 8A). Levels of phospho-4EBP were not significantly changed by the unloading or VX-745 treatment (Figure 8B). Levels of phospho-eEF2 were significantly increased in both HS and HSVX groups when compared with the control (Figure 8C). These results suggest that p38 signaling is not involved in the regulation of the unloading-induced p70S6K, 4EBP, and eEF2 phosphorylation.

## 3. Discussion and Conclusions

Unloading-induced skeletal muscle atrophy is a complex process. The molecular mechanisms involved in the regulation of the early stages of muscle atrophy are not well defined. Our study focused on the role of p38α MAP kinase in the regulation of early stages of unloading-induced muscle atrophy.

During initial stages of the study, we verified that VX-745 potently and specifically inhibited p38α MAP kinase under our experimental conditions. VX-745 inhibitor was highly specific for the p38α MAPK and it had minimal effects on other kinases. The majority of the previous publications used SB202190 p38 MAPK inhibitor [20,21]. Different to SB202190, VX-745 is a more potent inhibitor with an IC_50_ of 10 nM. SB203850 has an IC_50_ of 300–500 nM. Moreover, it was recently reported that SB202190 should not be used in research studies as an inhibitor of p38 MAPK due to its action on the autophagy–lysosomal axis [22]. Using a highly specific inhibitor, the current study showed that unloading-induced p38α MAP kinase phosphorylation was significantly reduced in the presence of VX-745 inhibitor.

The current study demonstrated that by inhibiting p38α MAP kinase phosphorylation, we were able to block unloading-induced muscle atrophy. Both absolute weight of soleus muscle and total body weight-adjusted weight of soleus muscle were decreased by unloading, and these decreases were blocked by the VX-745 treatment. It is well documented that early stages of unloading induce skeletal muscle atrophy [23], and that muscle atrophy is especially pronounced at the later stages of unloading [14,24]. Yuasa and colleagues previously showed that muscle-specific ablation of p38α MAP kinase diminished denervation-induced skeletal muscle atrophy [10]. The effect of p38α MAP kinase on calpains and ubiquitin-proteasome pathway was not evaluated in that study [10]. Total body weight was not statistically different in C, HS, and HSVX groups in our study. This suggests that 3 days of unloading do not have a significant effect on the total body weight. Moreover, it was previously reported that the weight of the soleus muscle is not affected by 2 days of fasting [25]. Therefore, a significant decrease of soleus muscle weight in the unloaded rats in our study was not affected by the food intake.

We previously showed that calpain-dependent breakdown of cytoskeletal proteins plays a critical role during early stages of unloading-induced proteolysis [9]. Calpains are activated by increased calcium ions in the cytoplasm [7]. We tested whether p38α MAP kinase is also involved in the regulation of unloading-induced calpain-dependent proteolysis. VX-745 treatment blocked the increase in calpain-1 expression during unloading. These data suggest that calpain-1 inhibition by VX-745 treatment could be involved in the observed in our experiments decrease of unloading-induced muscle atrophy.

The ubiquitin–proteasome pathway plays critical role in skeletal muscle atrophy. In our experiments, VX-745 treatment prevented increase of the ubiquitin expression during unloading. Expression of muscle-specific E3 ubiquitin ligases MAFbx and MuRF1 is activated shortly after unloading and peaks at the third day [16,26]. VX-745 treatment prevented unloading-induced increase in MuRF-1, whereas MAFbx expression was not affected. It was previously suggested that MuRF-1 and MAFbx could play different roles in the atrophic process. MuRF-1 is mostly involved in the breakdown of myofibrillar proteins [27], whereas MAFbx controls both the breakdown of cytoskeletal proteins as well as protein synthesis [28].

It was previously reported that p38 regulates muscle atrophy [10]. The p38 MAP kinase family has four members (α, β, γ, and δ), and this enables a large variety of responses when activated by different extracellular signals. In particular, it was shown that p38β, but not p38α, is mediating muscle catabolism by activation of C/EBPβ and upregulation of MAFbx [29]. In mice with muscle-specific ablation of p38α MAP kinase, the expression of MuRF1 and MAFbx in fast muscle was significantly lower than in wild-type mice [10]. This correlates well with our findings, suggesting that p38α MAPK is involved in the regulation of MuRF-1 mRNA expression upon rat soleus muscle unloading.

Previous studies suggested that unloading-induced upregulation of MuRF1 and MAFbx expression was controlled by the transcription factor FoxO [6]. The level of FoxO3 phosphorylation in our study was reduced only in the HS group, but not in the HSVX group. This implies that changes in MuRF-1 expression in our study could be controlled by FoxO3. Our data correlate with previous studies, showing that FoxO3 activity can be regulated via p38α MAP kinase signaling. For example, transcriptional activity of FoxO3 was reduced in the muscle of mice with muscle-specific ablation of p38α MAP kinase [10]. p38 MAP kinase signaling also regulates nuclear translocation and transcriptional activity of FoxO3 in myoblasts [30]. It should be mentioned that, in addition to FoxO3, the expression of MuRF1 and MAFbx can be regulated by other transcription factors. This regulation of expression involves multiple mechanisms: phosphorylation, acetylation, and deacetylation. For example, when HDAC 1, HDAC 4, and HDAC 5 were inhibited in unloaded skeletal muscle of rats, this resulted in decreased expression of MAFbx, whereas MuRF1 expression was not changed [23]. Therefore, it is possible that in our current study the expression of MuRF1 and MAFbx could have been similarly regulated by multiple mechanisms. The role of Akt1 signaling as one of the key signaling pathways in skeletal muscle atrophy is well documented [31,32]. During muscle unloading, Akt1 promotes protein degradation through the mTOR and p70S6K pathways, induces expression of MuRF1 and MAFbx, and downregulates protein synthesis via dephosphorylation of FoxO transcriptional factor. In our experiments, Akt1 phosphorylation was decreased in both HS and HSVX groups. At the same time, the level of FoxO3 phosphorylation was reduced only in the HS group, but not in the HSVX group. This observation implies that Akt1 signaling probably does not have a significant effect on the FoxO3 phosphorylation in the HSVX group.

Transcriptional coactivator PGC-1α also plays an important role during skeletal muscle atrophy [33]. It was reported that the level of PGC-1α expression was decreased after 3 days of skeletal muscle unloading [18]. Upstream signaling pathways regulating PGC-1α expression are not well elucidated. It was suggested that calcineurin A, CaMK, p38 MAPK, and AMPK pathways may be involved in the regulation of PGC-1α [34,35,36]. In agreement with previous observations, our data also showed that unloading decreased PGC-1α protein expression. Treatment with VX-745 inhibitor prevented unloading-induced decrease in PGC-1α protein content. On the basis of these results, we conclude that p38α MAP kinase signaling is important for the regulation of PGC-1α expression during unloading. PGC-1α overexpression decreases expression of E3 ubiquitin ligases [18]. In our study, unloading-increased expression of MuRF1 E3 ubiquitin ligase correlated with a decrease in PGC-1α protein content. Treatment with VX-745 inhibitor blocked both MuRF1 increase and the decrease of PGC-1α protein content. This suggests that PGC-1α can be involved in the regulation of MuRF1 expression during early stages of unloading-induced skeletal muscle atrophy. PGC-1α expression is downregulated by IL-6 signaling [19]. Chronic IL-6 injections into skeletal muscle results in muscle atrophy [37]. Muscle unloading increases IL-6 expression [33], whereas inhibiting IL-6 during unloading diminishes MuRF1 activation without any effect on the MAFbx expression [38]. IL-6 expression in myotubes is regulated by p38 MAPK [39]. In our experiments, unloading increased expression of IL-6 and IL-6 receptors, whereas treatment with VX-745 inhibitor blocked/diminished this increase. This is consistent with previously published data. The unloading-induced decrease in PGC-1α expression correlates with the increase in IL-6 expression, suggesting that IL-6 may be involved in the regulation of PGC-1α expression in a p38α MAPK-dependent manner.

Protein synthesis offsets protein degradation and helps to maintain muscle mass during unloading. Skeletal muscle protein synthesis is regulated by a number of signaling pathways, including eEF2, p70S6k, and 4E-BP1 phosphorylation. Our previous studies showed increased p70S6k phosphorylation during early staged of unloading [40]. Akt1 and p70S6k phosphorylation can be regulated by rapamycin-sensitive mTORC1 signaling as well as by mTORC2-signaling pathway [41,42]. We showed that p70S6k phosphorylation was increased by unloading, and inhibiting p38α MAP kinase had no effect on this increase. We conclude that p38α MAP kinase is not involved in the regulation of p70S6k phosphorylation at the early stages of unloading. The levels of total and phospho-4E-BP1 were not changed in response to unloading and were not affected by the treatment with VX-745 inhibitor. It is likely that 4E-BP1 and p70S6k phosphorylation in skeletal muscle is regulated via different signaling pathways [43]. On the basis of these data, it is possible that 4E-BP1 phosphorylation did not play an important role in muscle atrophy in our study.

It was previously reported that eEF2 phosphorylation suppresses eEF2 binding to the ribosome and inhibits polypeptide chain growth [44]. eEF2 phosphorylation is regulated by calcium concentrations, low pH value, activities of protein kinase A, and AMP-activated protein kinase [45,46,47], which leads to the inhibition of elongation. In our experiments, eEF2 phosphorylation was increased by unloading and was not regulated by the treatment with VX-745 inhibitor, whereas unloading-induced muscle atrophy was blocked in the HSVX group. We conclude that eEF2 phosphorylation is not regulated by p38α MAP kinase, and the preservation of muscle mass is not a result of improved protein elongation or translation.

In summary, in our experiments inhibition of p38α MAP kinase using VX-745 inhibitor prevented unloading-induced skeletal muscle atrophy. Muscle atrophy was mostly regulated by calpain-1 and E3 ubiquitin ligases, whereas protein translation and elongation were not crucially important. The current study, for the first time, showed that p38α MAP kinase signaling regulates unloading-induced increase in MuRF1, but not MAFbx expression. This regulation might involve IL-6 and PGC-1α as downstream signaling molecules.

## 4. Methods and Materials

### 4.1. Animal Procedures

The experiments were performed in compliance with the internationally accepted regulations and rules of biomedical ethics. The study was approved by the Committee on Bioethics of the Russian Academy of Sciences (protocol 481, 06/12/2018). All animal experiments were performed at the Institute of Biomedical Problems, RAS, Russia. Animals were kept at 22 °C in a light-controlled environment (12:12 h light–dark cycle) with water and food available ad libitum. Twenty-four male Wistar rats (3 months old, 180–200 g body weight range) were obtained from the certified Nursery for the Laboratory Animals of the Institute of Bioorganic Chemistry of the Russian Academy of Sciences (Pushchino, Moscow region). Rats were randomly assigned to one of the three groups with eight animals per group: non-treated control (C), or three days of hindlimb suspension/unloading with (HSVX) or without (HS) p38α inhibitor VX-745 (10 mg/kg/day orally with small amount of food soaked in VX-745 solution; #A8686, APExBio, Houston, TX, USA). Care was taken to ensure that each rat consumed the entire small piece of food provided with each treatment. It was reported previously that drug delivery with food by voluntary ingestion might be a better option than stress-inducing gavage technique [48]. VX-745 (5-(2,6-dichlorophenyl)-2-(2,4-difluorophenylthio)-6H-pyrimido [1 ,6-b] pyridazin-6-one) is a highly potent (IC_50_ = 10 nM) inhibitor of p38α. VX-745 is also highly selective for p38α MAPK, with 20-fold selectivity over p38β and 1000-fold selectivity over the closely related kinases ERK1 and JNK1–3. The drug concentration was determined on the basis of the drug manufacturer’s protocol and previously published data [49], as well as current human clinical trials for dementia and Alzheimer’s disease (ClinicalTrials.gov). The control animals received equal amounts of food soaked in the vehicle solution. At the end of the 3-day experiment, rats were euthanized by overdose of 10% avertin solution (Sigma-Aldrich Corp., St. Louis, MO, USA) and soleus muscle was immediately dissected, weighed, divided into aliquots, frozen in liquid nitrogen, and stored at -85°C for the subsequent analyses.

### 4.2. Hindlimb Suspension Protocol

The tail-traction method of noninvasive tail-casting procedure was used for the hindlimb suspension, as previously described [8]. Suspended animals were free to move around the cage using their forelimbs to obtain food and water.

### 4.3. Protein Extraction and Western Blot Analysis

Total protein extracts were prepared from 50 mg of frozen soleus muscle. Complete Protease Inhibitor Cocktail (#sc-29130, Santa Cruz Biotechnology, Dallas, TX, USA), Phosphatase Inhibitor Cocktail B (#sc-45045, Santa Cruz Biotechnology, Dallas, TX,, USA), PMSF (1 mM), aprotinin (10 µg/mL), leupeptin (10 µg/mL), and pepstatin A (10 µg/mL) were used to maintain extract integrity and function. Quick Start Bradford Protein Assay (Bio-Rad Laboratories, Hercules, CA, USA) was used to quantify protein content. The samples were diluted in Laemmli buffer, run on 10% SDS-PAGE (20 µg/lane), and transferred to a nitrocellulose membrane (Bio-Rad Laboratories, Hercules, CA, USA). Membranes were blocked with blocking buffer (5% nonfat milk powder, TBS pH 7.4, and 0.1% Tween-20) and incubated overnight at 4°C with the primary antibodies. We used primary antibodies against total p38 MAPK (1:1000; #9212, Cell Signaling Technology, Danvers, MA, USA) and phosphorylated p38 MAPK (Thr180/Tyr182; 1:1000; GTX59567, GeneTex, Irvine, CA, USA), total Akt (1:1000; #2920, Cell Signaling Technology, USA) and phosphorylated Akt (Ser 473; 1:1500, #4058, Cell Signaling Technology, USA), phosphorylated eEF2 (Thr56; 1:1000; #2331, Cell Signaling Technology, Danvers, MA, USA), total eEF2 (1:1000; #2332, Cell Signaling Technology, Danvers, MA, USA) and phosphorylated 4E-BP1 (Thr37/46; 1:1000; #2855, Cell Signaling Technology, Danvers, MA, USA), total 4E-BP1 (1:1000; #9452, Cell Signaling Technology, Danvers, MA, USA), total FoxO3 (1:1000; #2497, Cell Signaling Technology, Danvers, MA, USA) and phosphorylated FoxO3 (Ser 253; 1:1000; #sc-101683, Santa Cruz Biotechnology, Dallas, TX, USA), PGC-1α (1:3000, ab54481, ABCAM, Cambridge, MA, USA USA), and MuRF-1 (1:500; #183094, ABCAM, Cambridge, MA, USA). Blots incubated with antibodies against GAPDH (1:10000, #G041, Applied Biological Materials Inc., Richmond, BC, Canada) were used for the normalization of loading. After three washes (10 minutes each) with TBS-Tween (TBS and 0.1% Tween-20), the membranes were incubated for 1 hour at room temperature with horseradish peroxidase-conjugated goat anti-rabbit (1:30,000, #111-035-003, Jackson Immuno Research, West Grove, PA, USA) or goat anti-mouse (1:25,000, #5178-2504, Bio-Rad Laboratories, Hercules, CA, USA) secondary antibodies. The membranes were washed again in TBS-Tween three times, incubated with Immun-Star HRP (horseradish peroxidase) Chemiluminescent system (Bio-Rad Laboratories, Hercules, CA, USA). All images were analyzed within the linear range. The protein bands were quantified using a C-DiGit Blot Scanner (LI-COR Biotechnology, Lincoln, NE, USA) and Image Studio C-DiGit software. The control and experimental samples were run on the same gel. Total protein staining (Ponceau S) was used as loading controls (data not shown). GAPDH protein content used for the normalization was not changed between the groups. The protein expression data for each group are expressed as a percentage of the control group values.

### 4.4. RNA Isolation and Reverse Transcription

Total RNA was extracted from 10 mg of frozen soleus muscle samples using an RNeasy Micro Kit (Qiagen, Hilden, Germany). RNA samples were treated with proteinase K and DNase I. RNA concentration was evaluated using a UV 2450 spectrophotometer (Shimadzu, Japan). Isolated RNA in aqueous solution was frozen at −85°C for storage. Reverse transcription was performed by incubating 0.5 µg of RNA, random hexamers d(N)6, dNTPs, RNase inhibitor, and MMLV (Moloney Murine Leukemia Virus) reverse transcriptase for 60 min at 42 °C.

### 4.5. Quantitative PCR Analysis

One microliter of cDNA was amplified in a 25 µL SYBR Green PCR reaction containing 1× Quantitect SYBR Green Master Mix (Syntol, Moscow, Russia) and 10 *p*M of each forward and reverse primer. Sequences of the primers used in the current study were as follows: forward 5′-CTACGATGTTGCAGCCAAGA-3′ and reverse 5′-GGCAGTCGAGAAGTCCAGTC-3′ for MAFbx; forward 5′-GCCAATTTGGTGCTTTTTGT-3′ and reverse 5′-AAATTCAGTCCTCTCCCCGT-3′ for MuRF-1; forward 5′-CACCAAGAAGGTC AAACAGGA-3′ and reverse 5′-GCAAGAACTTTATTCAAAGTGCAA-3′ for ubiquitin, forward 5′-ACGGCAA GTTCAACGGCACAGTCAA-3′ and reverse 5′-GCTTTCCAGAGGGGCCATCCACA-3′ for GAPDH; forward 5′ TCATGAAGTGTGACGTTGACATCC-3′ and reverse 5′-GTAAAACGCAGCTCAGTAACAGTC-3′ for β-actin.

The annealing temperature was set on the basis of the optimal annealing temperature of PCR primers. The amplification was monitored in real-time using a iQ5 Multicolor Real-Time PCR Detection System (Bio-Rad Laboratories, Hercules, CA, USA). To confirm the amplification specificity, PCR products from each primer pair were subjected to a melting curve analysis. Relative quantification was performed on the basis of the threshold cycle (CT value) for each PCR sample [50]. Initially, two housekeeping genes were evaluated for normalization: GAPDH and β-actin. Normalization to the level of expression of GAPDH and β-actin showed similar results (data not shown). GAPDH was chosen for the normalization of all quantitative PCR analysis experiments in the current study.

### 4.6. Statistical Analysis

All PCR data are expressed as median and interquartile range (0.25–0.75). Statistical analysis was performed using the REST 2009 v.2.0.12 (Qiagen, Hilden, Germany) and Origin Pro v.8.0 (OriginLab Corp., Northampton, MA, USA) programs. All Western blot data are expressed as means ± SE. Significant differences between groups were statistically analyzed using two-way ANOVA followed by Tukey’s test. When normality testing failed, data were analyzed by nonparametric methods (Kruskal–Wallis ANOVA followed by Dunnett’s test). Differences with values of *p* < 0.05 were considered statistically significant.
